# Redefining the Australian Anthrax Belt: Modeling the Ecological Niche and Predicting the Geographic Distribution of *Bacillus anthracis*

**DOI:** 10.1371/journal.pntd.0004689

**Published:** 2016-06-09

**Authors:** Alassane S. Barro, Mark Fegan, Barbara Moloney, Kelly Porter, Janine Muller, Simone Warner, Jason K. Blackburn

**Affiliations:** 1 Spatial Epidemiology and Ecology Research Laboratory, Department of Geography, University of Florida, Gainesville, Florida, United States of America; 2 Emerging Pathogens Institute, University of Florida, Gainesville, Florida, United States of America; 3 AgriBio, Centre for Agribiosciences, Biosciences Research, Department of Economic Development, Jobs, Transport and Resources, Bundoora Victoria, Australia; 4 New South Wales Department of Primary Industries, Biosecurity Intelligence and Traceability, Orange New South Wales, Australia; 5 Chief Veterinary Officer's Unit, Department of Economic Development, Jobs, Transport and Resources, Attwood Victoria, Australia; University of Tennessee, UNITED STATES

## Abstract

The ecology and distribution of *B*. *anthracis* in Australia is not well understood, despite the continued occurrence of anthrax outbreaks in the eastern states of the country. Efforts to estimate the spatial extent of the risk of disease have been limited to a qualitative definition of an anthrax belt extending from southeast Queensland through the centre of New South Wales and into northern Victoria. This definition of the anthrax belt does not consider the role of environmental conditions in the distribution of *B*. *anthracis*. Here, we used the genetic algorithm for rule-set prediction model system (GARP), historical anthrax outbreaks and environmental data to model the ecological niche of *B*. *anthracis* and predict its potential geographic distribution in Australia. Our models reveal the niche of *B*. *anthrac*is in Australia is characterized by a narrow range of ecological conditions concentrated in two disjunct corridors. The most dominant corridor, used to redefine a new anthrax belt, parallels the Eastern Highlands and runs from north Victoria to central east Queensland through the centre of New South Wales. This study has redefined the anthrax belt in eastern Australia and provides insights about the ecological factors that limit the distribution of *B*. *anthracis* at the continental scale for Australia. The geographic distributions identified can help inform anthrax surveillance strategies by public and veterinary health agencies.

## Author Summary

This study explores the spatial ecology of *Bacillus anthracis*, the causative agent of anthrax disease, in Australia. Globally, anthrax is a neglected zoonotic disease that primarily affect herbivores and incidentally humans and all warm-blooded animals. Here, we used historic anthrax outbreaks for the period 1996–2013 and environmental factors in an ecological niche modelling framework to quantitatively define the ecological niche of *B*. *anthracis* using a genetic algorithm. This was projected onto the continental landscape of Australia to predict the geographic distribution of the pathogen. The ecological niche of *B*. *anthrac*is is characterized by a narrow range of ecological conditions, which are geographically concentrated in two disjunct corridors: a dominant corridor paralleling the Eastern Highlands runs from north Victoria to central east Queensland through the centre of New South Wales, while another corridor was predicted in the southwest of Western Australia. These findings provide an estimate of the potential geographic distribution of *B*. *anthracis*, and can help inform anthrax disease surveillance across Australia.

## Introduction

Anthrax is a zoonotic disease caused by *Bacillus anthracis*, an aerobic, gram-positive spore-forming bacterium. *Bacillus anthracis* primarily affects herbivores; though most warmed-blooded mammals may be susceptible [1], including humans. Anthrax is an ancient disease that has caused losses of livestock and wildlife populations prior to and throughout the 20^th^ century and remains enzootic with seasonal variations in many parts of the world [2, 3]. Transmission remains poorly understood, but ingestion of spores is the dominant hypothesis for herbivores [4]. Grazing mammals (e.g. cattle, sheep, zebras) can be infected by ingesting spores present in contaminated soils, while browsers (e.g. deer) may also ingest the pathogen with contaminated foliage [5, 6]. Biting flies may be involved in transmission on some landscapes [7, 8] and inhalation cannot be ruled out [9]; each mechanism requires further study. In each case, transmission is indirect and occurs where a susceptible host interacts with an environment that supports pathogen persistence. These environments can be characterized and mapped to define areas at risk for anthrax [5, 10].

The first recorded livestock anthrax in Australia dates to 1847 at Leppington, New South Wales where the disease slowly spread through cattle and sheep movements along stock routes [11]. In Victoria, anthrax was initially reported in the area around Warrnambool in the southwestern area of the state in 1886. From there, the disease apparently spread throughout the western districts of the state to Melbourne and elsewhere via the transport of infected sheep [12]. Historical records of livestock anthrax from the early 1900s to the 1920s indicate that the disease was more recurrent in New South Wales, where 80 confirmed anthrax outbreaks were recorded during that period [13]; twice as many as reported in Victoria during the same period. The 1930s saw an increase in livestock anthrax in Australia, especially in New South Wales and Victoria. For instance, in New South Wales, a total of 147 outbreaks were officially recorded from 1930 to 1936 [13], and about 200 during the period 1949–1962 with sheep most commonly infected, while an increase of incidence was observed in cattle in Victoria [12]. Additional outbreaks in Victoria in 1968 caused cattle and sheep deaths on 27 farms in the Yarrawonga/Shepparton area [14]. There is little published literature on anthrax in Australia during the period 1970–1990; though there were confirmed reports throughout NSW and Victoria.

**Fig 1:**
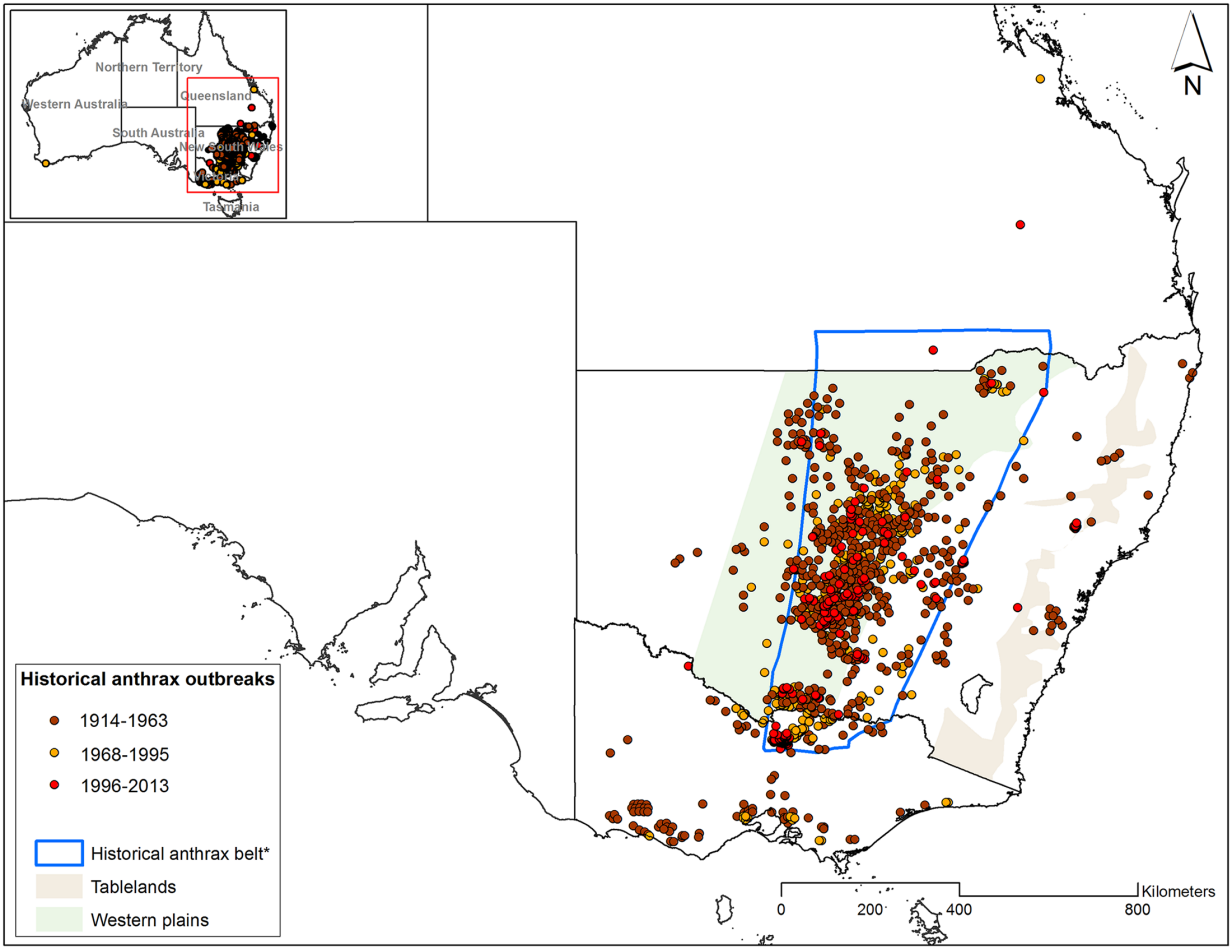
Spatial distribution of anthrax outbreak locations in Victoria, New South Wales and Queensland during three historical periods. The inset map shows one outbreak location in Western Australia. *Heads-up digitized from Durrheim et al.[17].

Summarizing the available Australian literature, the continent experienced overall reductions in the size and spatial distribution of livestock anthrax outbreaks across the latter half of the 20^th^ century. Similar patterns were documented in the United States [15] and the Ukraine [16]. The majority of anthrax outbreaks in recent decades have taken place across the Australian anthrax belt, which predominantly runs through the center of New South Wales [17]. The geography of the anthrax belt was originally described by Henry [13] and later roughly delineated by Allan [18] and Durrheim et al. [17] to map the extent of the endemic zone in Australia (Fig 1). This description of the anthrax belt is based solely on locations of disease incidents. The belt lies between the tablelands and the western plains in New South Wales, and reaches from northern Victoria at the southern extent, northward through New South Wales to the southern border of Queensland.

Occasionally, outbreaks have occurred outside of the historically defined anthrax belt. For example, an outbreak occurred in 2007–2008 in the Hunter Valley, a valley located ~350 km east of the belt. In that outbreak, 11 dairy farms in the Hunter Valley experienced unusual anthrax outbreaks in the summer of 2007 with clinical cases mainly observed in cattle. The last recorded livestock anthrax cases in the Hunter Valley prior to the 2007–2008 outbreaks occurred in 1939 [17].

An unprecedented livestock epizootic also occurred in the Goulburn Valley in the north of Victoria in the summer of 1997, affecting 83 dairy farms in the Stanhope/Tatura area. This was the largest anthrax epizootic reported in Australia since official reports of livestock anthrax began in 1914 in Victoria [19]. The Goulburn Valley in northern Victoria borders southernmost New South Wales and intersects the southern extension of the anthrax belt into Victoria [17]. Although the number of reported anthrax outbreaks within the anthrax belt has decreased in recent decades, outbreaks continue within and beyond its currently defined boundaries. Therefore, there is a need for ecological investigations of the distribution of *B*. *anthracis* and the identification of all potential risk zones in Australia to better inform anthrax surveillance.

Many environmental factors including climate and soil are known to prolong the survival of anthrax spores in the environment. Van Ness [20] postulated that suitable soils with high soil moisture, alkaline pH, and organic nutrients referred to as “incubator areas” may be conducive to the germination, vegetative growth and sporulation of *B*. *anthracis* independently of a mammal host. Recent experimental spore germination using a grass-soil model system also supported the possibility that this dynamic state occurs in the soil [21]. However, the study of Dragon et al. [22] demonstrated that in natural conditions, growth of *B*. *anthracis* outside a host leads to a rapid loss of virulence, and that vegetative forms cannot compete with other bacteria species in the soil. This latter study supports the “persistent spore theory” according to which, spores persist in the soil for very long periods of time until they come into contact with a susceptible host causing disease [23, 24]. Irrespective of which of these theories is correct, both recognize that soil is the natural reservoir of anthrax spores, which therefore implies that a greater understanding of the ecological conditions that allow spores to “persist” or “incubate” in the soil environment is essential for the prediction of the potential geographic distribution of *B*. *anthracis*.

Ecological niche modeling is one approach to estimate the potential geographic distribution of a species and has been applied to map *B*. *anthracis* habitat suitability for several landscapes [10, 25–29]. These approaches relate environmental covariates with historic occurrence data on the species (e.g. outbreak locations) using pattern matching genetic algorithms or statistical approaches [30]. Occurrence data are generally obtained from a subset of the landscape accessible by the species [31] and related to larger landscapes described by environmental covariates [5]. Broadly, ecological niche modeling techniques can be divided into presence-absence and presence-only approaches. In the former, the user provides both occurrence locations and locations where the species was not detected. In the latter, the modeling algorithm will exhaustively sub-sample pseudo-absence points from a user-defined amount of the sampling area or background, which has been recommended when spatial information on species’ absence is unavailable [32] or occurrence points derived from idiosyncratic data sources, as is common with historical disease data. Many ecological niche modeling studies have used the presence-only approach to successfully predict the potential geographic limits of organisms in disease ecology [25, 33–35], biogeography [36] and conservation biology [37] across spatial scales. Here we used a presence-only modeling approach to predict the geographic distribution of *B*. *anthracis* across Australia.

## Methods

### Anthrax occurrence data

**Fig 2:**
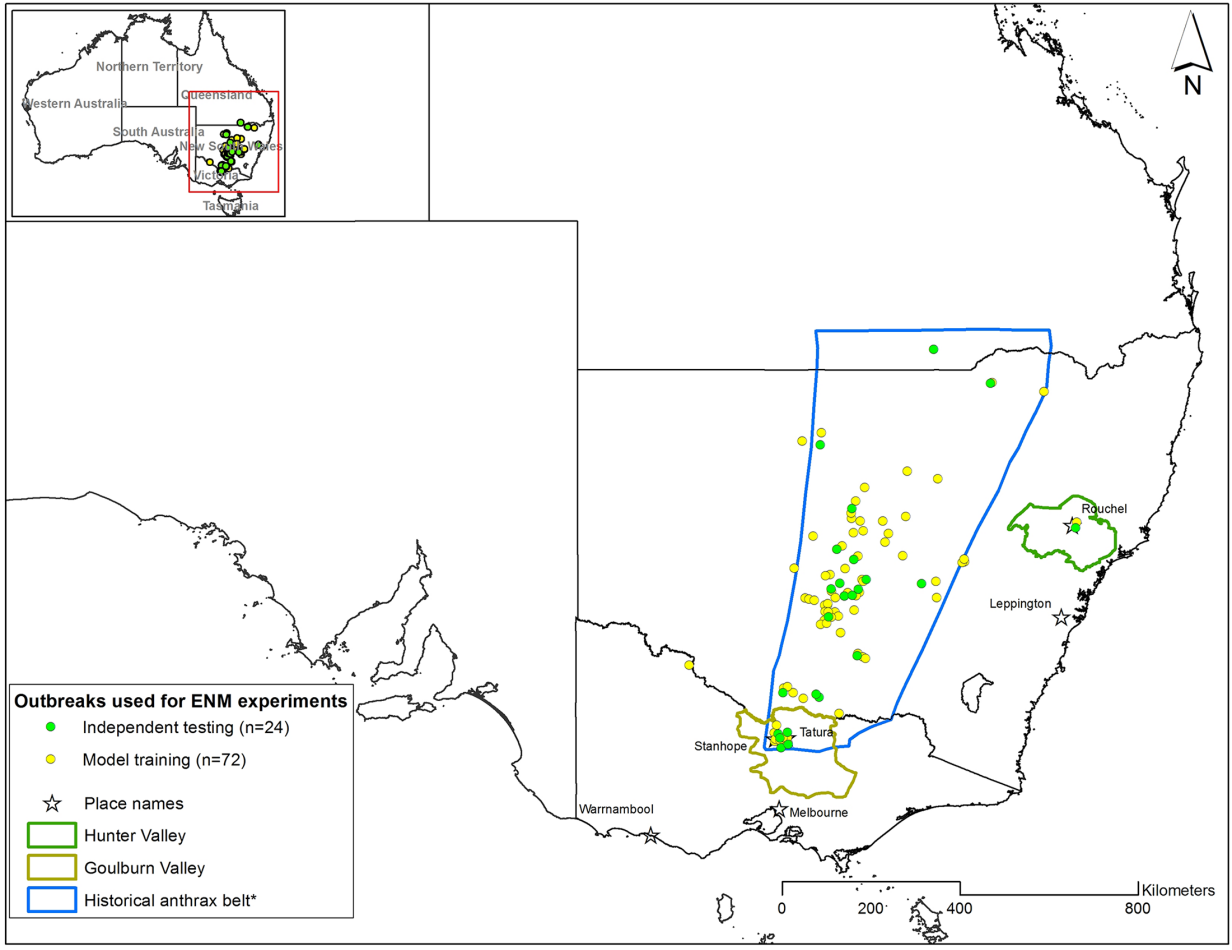
Distribution of anthrax outbreak locations used for ecological niche modeling (ENM) experiments. Yellow dots represent the data points used for model building (n = 72) and green dots were used for model validation (n = 24). *Heads-up digitized from Durrheim et al. [17].

A geographic information system (GIS) database of historical occurrence of livestock anthrax was constructed using anthrax locations heads-up digitized from Seddon and Albiston [12] and presence data provided by the Department of Economic Development, Jobs, Transport and Resources (DEDJTR) in Victoria, the Department of Primary Industries (DPI) in New South Wales and the Department of Agriculture, Fisheries and Forestry (DAFF) in Queensland, Australia (Fig 1). To ensure that all occurrence data were anthrax related deaths, only confirmed outbreaks (carcasses tested positive for *B*. *anthracis* or clinical confirmation), in the states of Victoria, New South Wales, and Queensland were retained for further analyses (Fig 2). An outbreak was defined as any location (infected farm or property) with one or more anthrax cases. Ideally, to predict the geographic distribution of *B*. *anthracis*, one would use occurrence data obtained from positive soil samples indicating the presence of the pathogen in the environment. Instead, outbreak locations were used as a proxy for *B*. *anthracis* occurrence data because anthrax-related death occurs after a relative short period of time following infection. For this study, we assumed that there were not great distances between infection source and carcasses. Additionally, in this study, *B*. *anthracis* infections and deaths occur on the same farms, and outbreak locations were represented by the geographic coordinates of infected farms. For each outbreak, the latitude and longitude were recorded along with additional attributes including date (day, month, and year), and total number of cases per animal species. Table 1 summarizes the spatial resolution and data collection methods for outbreaks for each state.

**Table 1:** Outbreak locality data used to develop the genetic algorithm for rule-set prediction (GARP).

Locations	Resolution	Geocoding	Number	GARP outbreaks		Date	Sources
				Training	Testing		
New South Wales	Farms	GPS coordinates	116	62	18	1996–2013	NSW DPI‡
	Property	Digitizing	223	0	0	1951–1963	Seddon and Albiston [12]
Victoria	Farms	GPS coordinates	86	10	5	1997–2009	DEDJTR†
	Farms	GPS coordinates	95	0	0	1968–1988	DEDJTR
	Property	Digitizing	91	0	0	1914–1963	Seddon and Albiston [12]
Queensland	Farms	GPS coordinates	1	0	0	1993	DEDJTR, DAFF#
	Farms	GPS coordinates	2	0	1	2002	DEDJTR, DAFF
Western Australia	Farms	GPS coordinates	1	0	0	1994	DEDJTR

҂ GPS: Global positioning systems

^†^ DEDJTR: Department of Economic Development, Jobs, Transport and Resources, Victoria;

^‡^ NSW DPI: Department of Protection Industries (DPI), New South Wales

^#^ DAFF: Department of Agriculture, Fisheries and Forestry, Queensland

Duplicate coordinates were removed from the database for ecological niche modeling experiments. We then filtered the database to include one outbreak location per 8x8 km pixel, the resolution of environmental data used for modeling (hereafter referred to as the spatially unique presence points) [26]. The ecological niche modeling algorithm, the genetic algorithm for rule-set prediction (GARP) utilizes a single point per pixel to indicate the presence of *B*. *anthracis*. Using more than one point per grid cell for model development is equivalent of using the same data for both the training and testing of a GARP model, which can lead to inflation of accuracy metrics [26].

### Environmental coverages

We used three groups of environmental coverages including bioclimatic (temperature and precipitation), edaphic (vegetation and soil properties), and topographic (altitude) factors known to influence the persistence of *B*. *anthracis* in the environment. Bioclimatic variables were downloaded at 30 arc-seconds (approximately 1x1 km spatial resolution) from the WorldClim website (http://worldclim.org) and are described in detail elsewhere [38]. Vegetation indices (8x8 km spatial resolution) were obtained from the Trypanosomiasis and Land Use in Africa (TALA) research group [39]. Soils data were extracted from the harmonized world soil database v1.2 (HWSD) available at the International Institute for Applied System Analysis (IIASA) (http://www.iiasa.ac.at) [40]. The HWSD data were available at 1x1km spatial resolution. The variables used for the ecological niche modeling are presented in Table 2.

**Table 2:** Environmental gradients used for GARP models of *Bacillus anthracis* in Australia.

Environmental variable (unit)	Variable name	Data source
Altitude (m)	alt	Worldclim†
Mean annual temperature (°C)	bio 1	Worldclim
Annual temperature range (°C)	bio 7	Worldclim
Annual precipitation (mm)	bio 12	Worldclim
Precipitation of the wettest month (mm)	bio 13	Worldclim
Precipitation of the driest month (mm)	bio 14	Worldclim
Average base saturation (%)	bs	HWSD*
Average calcium carbonate concentration (% weight)	caco3	HWSD
Average calcium sulfate concentration (% weight)	caso4	HWSD
Average soil pH (-log(H^+^))	pH(H_2_0)	HWSD
Average soil organic content (% weight)	OC	HWSD
TFA mean NDVI	wd0114a0	TALA‡
TFA NDVI annual amplitude	wd0114a1	TALA

^†^http://worldclim.org, [38]

* Trypanosomiasis and Land Use in Africa (TALA) research group, [39]

^‡^http://www.iiasa.ac.at, [40]

Correlated environmental variables were eliminated using a Pearson correlation test to retain the variables presented in Table 2, which were then clipped to the boundary of Australia. Since the environmental data were at different spatial resolutions (1x1 km and 8x8 km), all data layers were resampled to the coarsest cell size (8x8 km) using the GARP Datasets extension in ArcView 3.3 (Environmental Systems Research institute, Redlands, CA).

### Ecological niche modeling

In this study, we employed the genetic algorithm for rule-set prediction (GARP) and experiments were performed in DesktopGARP version 1.1.3 (DG). GARP is an expert-system, machine-learning algorithm that has been tested and widely used for species’ range prediction [32, 41–43]. Briefly, GARP develops a set of if/then logic string rules to relate observed occurrence data to environmental variables (bioclimatic, edaphic/substrate and topographic) [10]. Predicted presence or absence of a species within an ecological space are defined by one of four types of conditional rules including atomic, logit, and range or negated range rules [43]. Atomic rules use specific values or categories for each environmental variable (e.g. IF temperature = [35°C] AND precipitation = [325 mm] AND pH = [8.5] AND ndvi = [0.5] THEN species = PRESENCE/ABSENCE). Logit rules are fitted logistic regression functions (e.g. IF temperature*0.0078—precipitation*2.5 + pH*0.0039 + ndvi*0.0039 THEN species = PRESENCE). Upper and lower bounds for each environmental variable are specified in range rules (e.g. IF temperature = [14.6–19.5°C] AND precipitation = [348.25–757.51 mm] AND pH = [7.5–8] AND ndvi = [0.01–0.25] THEN species = PRESENCE). Negated range rules define conditions outside of variable ranges (e.g. IF NOT temperature = [15.5–28°C] AND precipitation = [143–1693 mm] AND pH = [6.5–8] AND ndvi = [0.25–0.45] THEN species = ABSENCE). The rules are developed through evolutionary refinement by testing and selecting rules on random draws of presence points from known occurrences data and pseudo-absences localities generated internally from the wider study area. A one-tailed significance χ^2^ test is then calculated in order to evaluate the quality of a rule at predicting the ecological distribution (presence or absence) of the species [43]. The stochastic process of deriving and evolving rules results in random walks through variable space resulting in multiple models. Each model is a set of 50 presence/absence rules that are projected onto the geographic landscape to estimate the potential geographic distribution of the species as a binary output (absence = 0, presence = 1).

### Model building and evaluation

Spatially unique presence points (N = 96 anthrax outbreak locations) were partitioned into training and independent test datasets for model building and evaluation. The geospatial modeling environment (GME, www.spatialecology.com) was used to randomly select 75% of the occurrence data points (n = 72) for models building and 25% of the points (n = 24) for calculating accuracy metrics [44–46]. To evaluate the effects of randomly sub-setting presence points, the selection process was repeated 10 times to develop 10 different GARP experiments. For each experiment, we ran up to 200 models with a maximum of 1,000 iterations and a convergence limit of 0.01. We allowed GARP to internally partition training data into a 75%/25% for model development and rule selection. We used the best subset procedure to select the best 20 models under a 10% hard omission threshold and a 50% commission threshold. Those 10 best subset models from each GARP experiment were then imported in ArcMap and summated using the raster calculator tool in the Spatial Analyst extension. The resulting composite raster layer, with pixel values ranging from 0 to 10, is a surface depicting the potential geographic distribution of *B*. *anthracis* in Australia. The higher the pixel values, the greater the potential that the environmental conditions will support pathogen persistence [25]. Model agreements from 0 to 5 were reclassified as not suitable and those greater or equal to 6 were considered most suitable to support *B*. *anthracis* persistence [26].

Model accuracy for each GARP experiment was calculated with the 25% independent testing data withheld from model building. Three metrics were used to measure accuracy: the area under curve (AUC) in a receiver operating characteristic (ROC) analysis, omission (a measure of false negatives) and commission (the proportion of the landscape falsely predicted as present) [44, 47]. The AUC was used to evaluate the overall performance of each composite predictive model (10-best subset model). An AUC of 0.5 indicates a random model whereas an AUC of 1 suggests a perfect model [42, 47]. Total omission was calculated as the percent of independent test points predicted absent by the composite predictive model and the average omission as the average omission across each of the 10 best models. Total and average commissions are the percent of pixels predicted as presence by the composite predictive model and the average of this value for the 10 best models, respectively [48].

Overall predicted area and the accuracy metrics were used to rank the 10 GARP composite predictive models. The best composite predictive model with the higher AUC value and lower omission error was retained to describe the potential geographic distribution of *B*. *anthracis* for Australia (S1 Fig) and to perform the rule-set analysis.

### Analysis of environmental parameters

Rule types from each of the 10 best models in the highest ranked experiment were extracted using a python script (K.M. McNyset, US NOAA) to illustrate the relative number of each rule type [35]. From each rule-set, dominant rules that cumulatively predicted over 90% of the landscape were also identified in order to extract maximum and minimum values of the environmental variables of each presence rule type. The median of minimum and maximum values for each covariate in a given rule were calculated in Microsoft Excel 2010 and plotted as bar graphs to illustrate the ranges of each covariate [49].

## Results

Each GARP experiment reached convergence of accuracy (0.01) prior to the maximum 1000 iterations. The accuracy metrics of all ten GARP experiments are ranked and summarized in S1 Table. Metrics indicated all ten experiments were accurate and predicted highly similar geographic distributions. The potential geographic distributions of *B*. *anthracis* predicted by the 10 GARP experiments are illustrated in S1 Fig. Experiment number 5 had the highest AUC score and lowest omission errors; its AUC score was 0.966 and significantly different from a line of no information (p<0.01). And the total and average omission errors were 0.00% and 0.83% respectively, meaning that all independent testing data were correctly predicted by each of the 10 models in the best subset. The total and average commission for experiment number 5 were 6.24% and 12.15% of the landscape, respectively (Table 3).

**Table 3:** Accuracy metrics of the GARP experiment 5 for predicting the ecological niche model of *Bacillus anthracis* in Australia.

Metric	Model specifications
N to build models‡	72
N to test models	24
Total omission	0.00
Average omission	0.83
Total commission	6.24
Average commission	12.15
AUC*	0.966
SE	0.026
Z	11.37

^‡^N was divided into 75% training/25% testing for each model iteration

* AUC = Area under the curve

**Fig 3:**
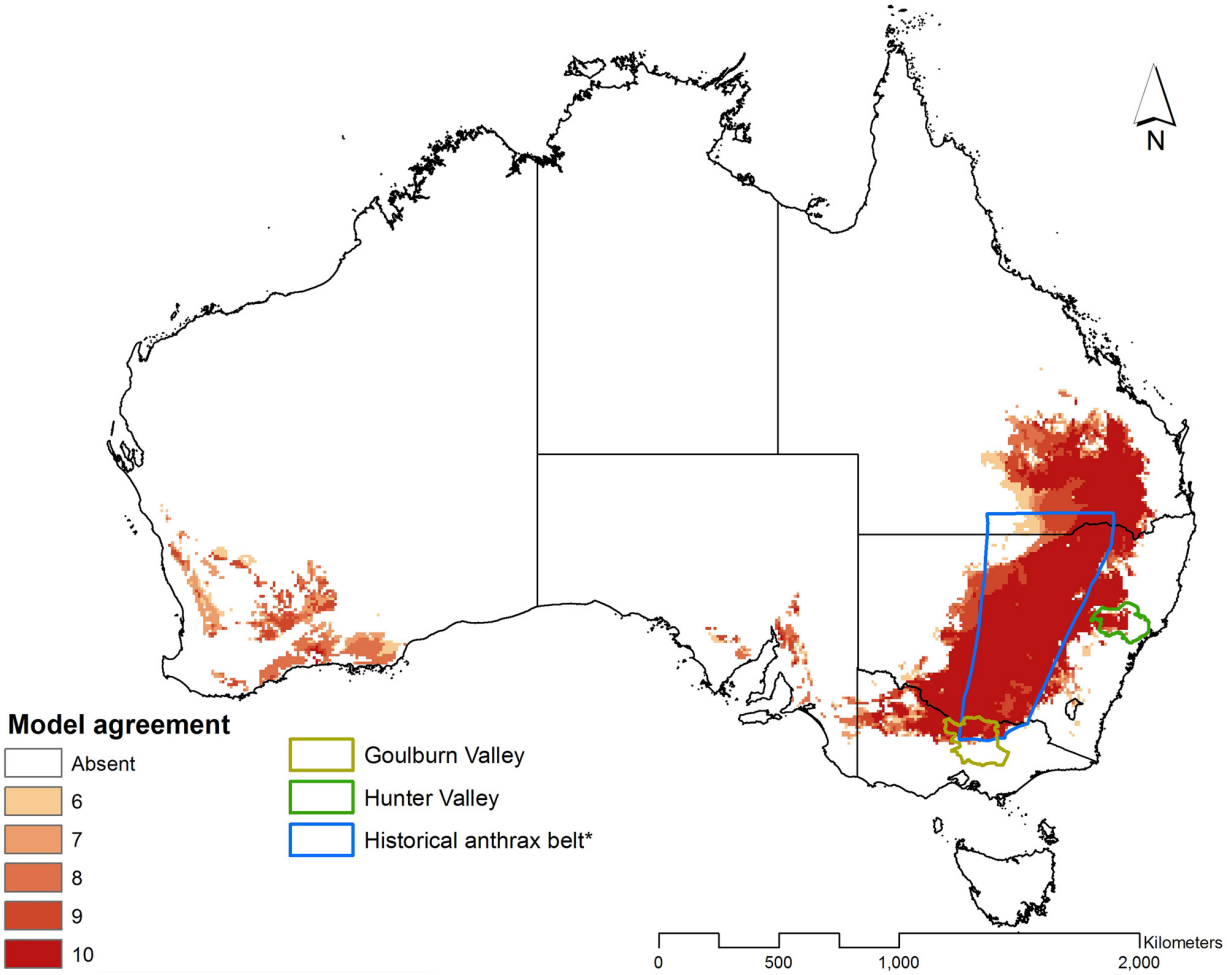
Predicted geographic distribution of *Bacillus anthracis* in Australia based on GARP ecological niche modeling. Model agreements represent the number of models in the best subset of models predicting the area to be conducive to *Bacillus anthracis* persistence. *Heads-up digitized from Durrheim et al.[17].

Broadly, experiment number 5 predicted areas that stretch from north Victoria to northeast Queensland and running parallel with the eastern coastal region of Australia (Fig 3). The predicted areas also expand from northwest Victoria into small areas in the south of South Australia. In the southern part of Western Australia, the predicted geographic space of *B*. *anthracis* spans an area from the south to the southwest of the state. The interior of the country and the state of Tasmania were not predicted to be suitable for *B*. *anthracis* persistence (based on the conservative criteria of 6 or more models in a best subset).

S2 Table summarizes the rule types, number and proportion of each of the 10 best subset models from GARP experiment 5. Range rules represented 97.8% of the rules in rule-set, whereas negated rules accounted for only 1.8%. There were only 2 logit rules kept in experiment 5. There were no atomic rules.

**Fig 4:**
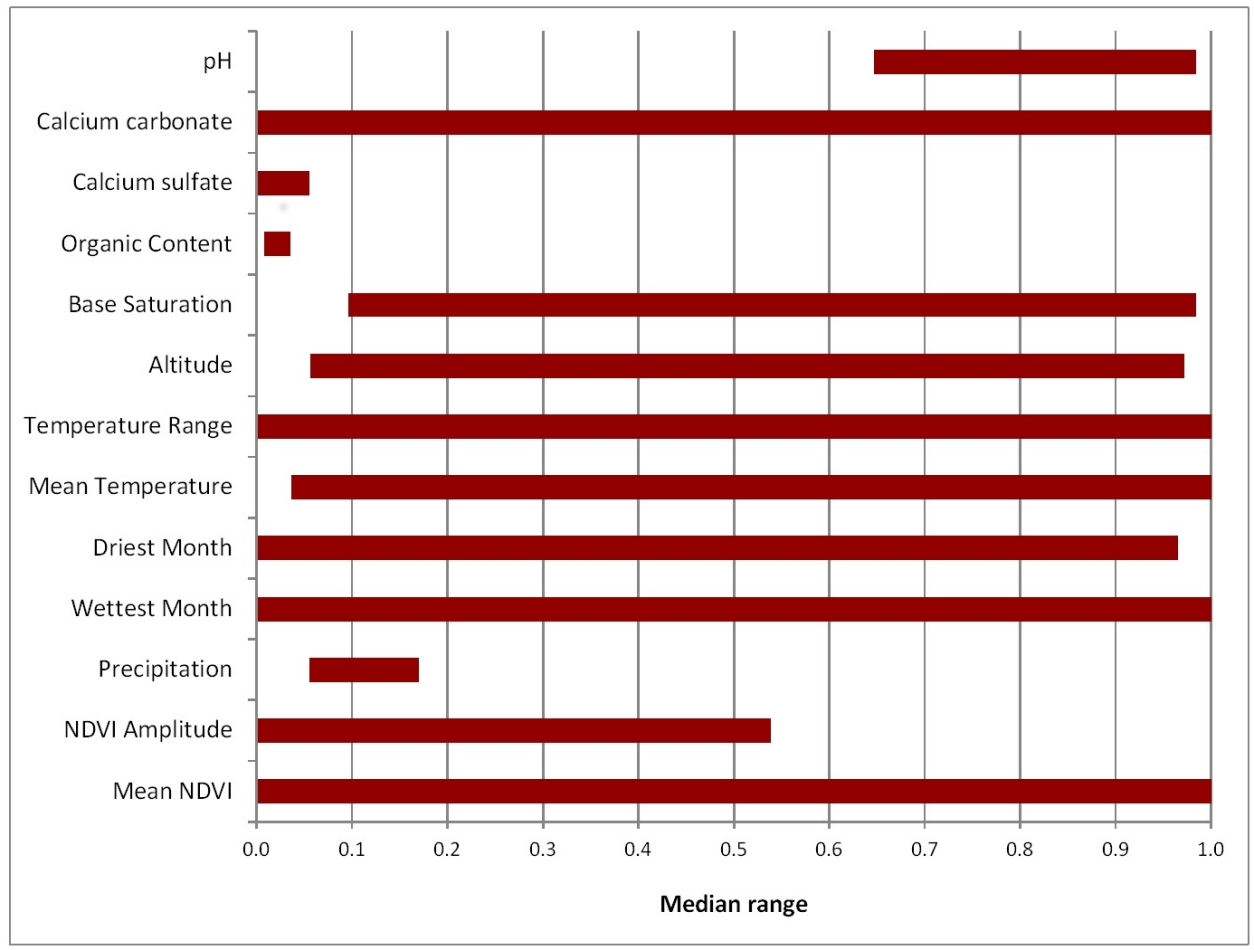
Median range of environmental variables extracted from the dominant rules in the best subset of models in a GARP experiment. The most limiting variables associated with the potential geographic distribution of *Bacillus anthracis* in Australia are represented by narrower median ranges.

Fig 4 illustrates narrow median range values for the following environmental variables: soil pH, calcium sulfate, organic content, and annual precipitation.

## Discussion

This study aimed to improve our understanding of the landscape ecology of *B*. *anthracis* and to predict the geographic distribution of the pathogen across Australia. We revised the geographic extent of the historical anthrax belt [17] that was defined by reported outbreaks and did not explicitly consider ecological conditions. Here we modeled the geographic distribution of *B*. *anthracis* based on environmental covariates known to be correlated with pathogen persistence enabling a quantitative redefinition of the anthrax belt. The distribution of *B*. *anthracis* has long been associated with environmental factors including soil and climatic parameters [20, 22, 50]. Incorporating these covariates into an ecological niche modeling framework provides a more accurate estimation of the geographic distribution of the pathogen, and therefore risk of anthrax, for Australia.

**Fig 5:**
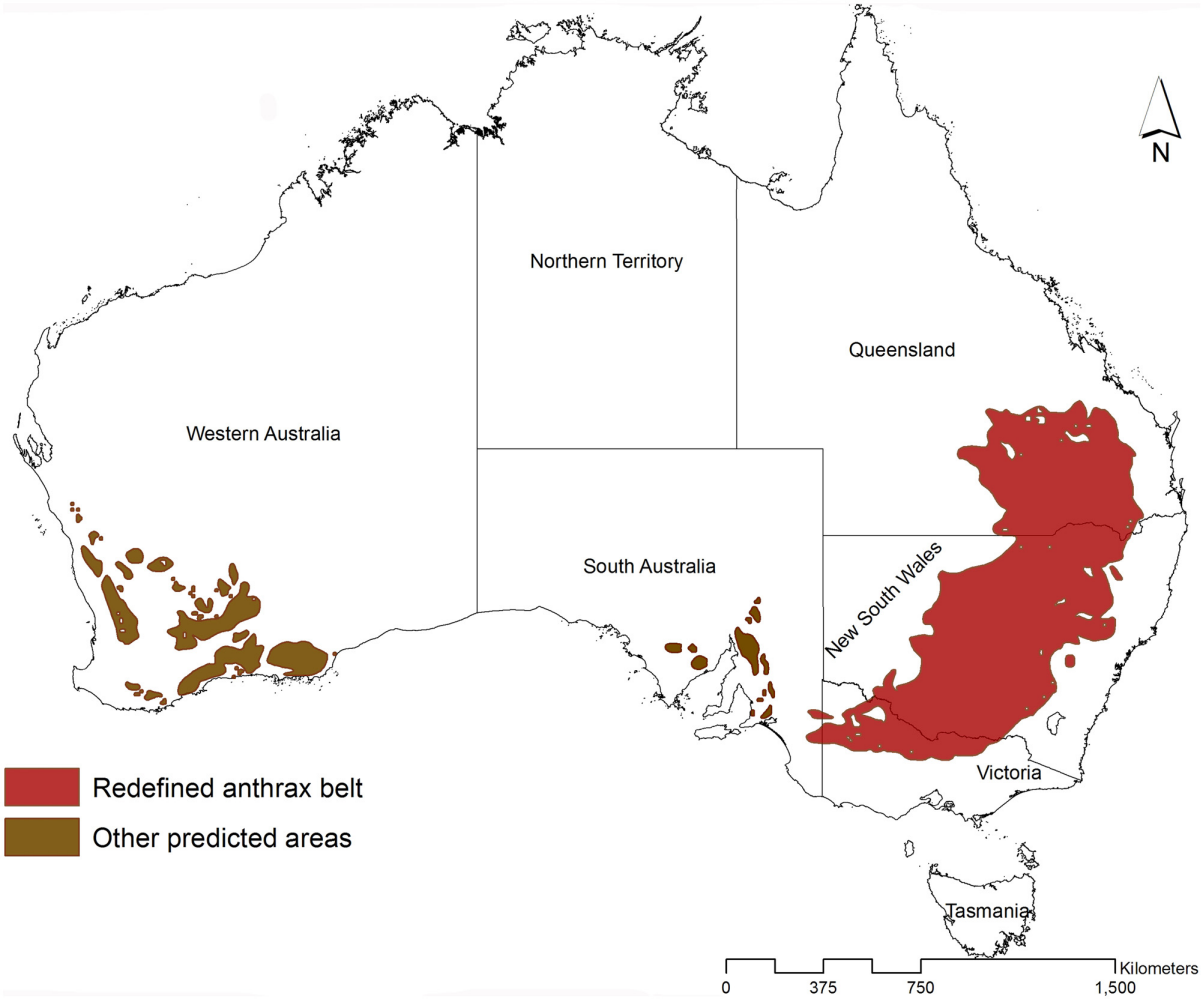
The Australian anthrax belt redefined using ecological niche modeling. Red areas define the extent of the anthrax belt, while khaki areas define regions, with few or no confirmed outbreaks in the historical record, which are predicted to potentially support the survival of *B*. *anthracis*.

The predicted areas of *B*. *anthracis* are distinctly separated into two anthrax zones: the southeast-northeast and southwest corridors (Fig 3). The southeast-northeast corridor, hereafter referred to as the ‘redefined anthrax belt’, parallel the Eastern Highlands, stretching from north Victoria to central eastern Queensland through New South Wales where it traverses the western region of the Hunter Valley. The redefined anthrax belt extends far beyond Durrheim et al. [17] and captures many of the historical anthrax locations (Figs 5 and S2). In Victoria, the models also predicted the northern area of Goulburn Valley. This prediction includes South Australia along the Spencer Gulf on the southern coast of Australia in an area disjunct from the redefined anthrax belt. In a second disjunct area in Western Australia, the models predict part of the Nullarbor Plain on the Great Australian Bight coast, and the Darling Range in the Perth area.

The rule-set analysis indicated that the predicted ecological niche of *B*. *anthracis* is defined by a narrow range of high soil pH, low organic content, calcium sulfate, and annual precipitation (Fig 4). Across the best subset, a single rule per model captured nearly all of the predicted presence (S3 Fig). This is in contrast to the models developed for the United States, where presence rules captured presence with rules delineating eastern or western conditions [15] or presence rules in Kazakhstan dominated by northern and southern rules [49].

Historically, livestock anthrax was widespread in Australia, in particular Victoria and New South Wales. A comparison of past (1914–1963 and 1968–1995; Table 4) and recent epizootics (1996–2013; included in the model building process) confirmed a decrease in the number and spatial extent of anthrax outbreaks in the affected states, New South Wales and Victoria. This decrease is most likely due to the implementation of improved surveillance measures, livestock vaccination, the destruction of infected carcasses by burning, site decontamination and quarantine of affected livestock and properties [13]. A similar pattern of decrease of anthrax incidence was observed in the United States [15] and Ukraine [16]. In the United States, the use of an efficacious vaccine, along with better anthrax disease management strategies also resulted in a decrease in number of reported endemic counties from the 1950s onwards. In Ukraine, mass vaccination campaigns and effective control measures (burning of contaminated carcasses and sites decontamination) resulted in a reduction of anthrax foci from the early 1970s to the post-soviet period (1991 to the present day) [16].

**Table 4:** Proportion of historical outbreaks mapped but not used for ENM experiments that occur within the predicted space of the redefined Australian anthrax belt.

State	Years	Number of outbreaks		Total	Percentage (%)
		Within	Outside		
Victoria	1996	0	0	0	0.00
	1968–1995	78	17	95	82.10
	1914–1963	19	72	91	20.87
	1914–1996	97	89	186	52.15
NSW	1996	9	1	10	90.00
	1968–1995	301	4	305	98.68
	1914–1963	458	46	504	90.87
	1914–1996	768	51	819	93.77
Victoria-NSW-Queensland	1996	9	1	10	90.00
	1968–1995	379	21	400	94.75
	1914–1963	477	118	595	80.17
	1914–1996	865	140	1005	86.07

NSW: New South Wales

It has been reported that during the mid-19^th^ century, intensification of farming activities in Australia was associated with the use of unsterilized bone meal imported from India as a mineral supplement fed to livestock and as a fertilizer which led to the introduction of the pathogen [12]. The pathogen likely spread within Australia through the movement of diseased livestock along the southeast to northeast coastal corridor, and the contamination of stock routes with *B*. *anthracis* spores [12]. Contemporary livestock movement trajectories produced by the AusVet Animal Health Services [51] and East and Foreman [52] agree with historical livestock movements [12]. These movement trajectories perfectly intersect with areas of high model agreement.

The predicted geographic distribution of *B*. *anthracis* defines some suitable areas with no historical outbreak records, which may be due to over-prediction of the models. Nevertheless, it is worthwhile to note that over-predicting the geographic distribution of a species does not necessarily infer prediction error. The potentially over-predicted geographical distribution areas may represent an accurate illustration of the spatial extent of *B*. *anthracis* [53], despite the lack of presence records that could be used for testing the accuracy of our model in those areas. For example, using GARP, Blackburn et al. [25] successfully predicted suitable distributional areas for *B*. *anthracis* in the northwest corner of Montana, US, that had experienced anthrax outbreaks in 2005 although specific localities were unavailable for modeling. In South Australia, it has been reported that twenty three cattle died from anthrax in 1906 on a government farm at Islington [12], and six years later the disease also occurred in a metropolitan piggery at Unley after feeding pigs the carcasses of two horses, that had previously died from anthrax [12]. The source of infection at Islington was not mentioned, and the reported cases at Unley were not associated with direct contact to soil spores. However, these two areas overlap with the high agreement areas of our GARP models.

The models did not predict two outbreak locations that were withheld from model building, one in Western Australia and the other in Queensland. The anthrax cases in Western Australia were recorded in 1994 on three cattle properties in a localized area north of Walpole, where 29 cattle died from unknown sources from January to April 1994 [54]. In 1993, one cow died from anthrax on a grazing property near Rockhampton in Queensland, apparently from ingestion of contaminated feed [55]. In each case, the affected properties were outside of the predicted areas. Since anthrax is primarily a soil-borne disease, we hypothesize that these isolated cases, as well as the early outbreaks in the south coast areas of Victoria outside the predicted geographic distribution areas (S2 Fig), are likely attributable to causes other than ingestion of spores at grazing sites.

Anthrax was first recognized in Victoria in 1879 at Warrnambool followed by other areas in the south west of the state. The disease was later identified in southern and central Victoria following shipment of diseased sheep [12]. Seddon and Albiston [12] thought it is unlikely that the initial outbreak in the southwest of Victoria resulted from the spread of the disease from southern New South Wales, indicating that the introduction of the disease into this area came from other sources, followed by rapid spread over long distances to new areas by movement of stock by rail. The later distribution of the disease into the north of Victoria is considered to be most probably due to stock traveling over the border from NSW [12]. The distribution of anthrax throughout Victoria has changed over time with the majority of outbreaks post-1968 falling within the predicted zone and those prior to 1968 falling outside of this zone (Table 4). We hypothesize that the presence of disease incidents along the south coast of Victoria outside of the predicted geographic distribution prior to 1968 may represent constant reintroductions of the disease into these areas, given their proximity to ports and transport routes combined with possible short term survival and local spread.

This study redefines the anthrax belt of Australia, which is presently defined by the location of anthrax cases, by integrating ecological niche modeling and GIS. This approach provides insights about the ecological factors that limit the distribution of *B*. *anthracis* at the continental scale for Australia. The geographic distributions presented here can help inform anthrax surveillance strategies by public and veterinary health agencies.

## Supporting Information

S1 Fig Predicted geographic distributions of *Bacillus anthracis* in Australia from 10 independent GARP experiments. Geographic distributions are ranked from top left corner to right based on the highest AUC and lowest total omission rates listed in S1 Table. Model agreements represent the number of model(s) predicting the area to be conducive to *Bacillus anthracis* persistence.
(TIF)

S2 Fig Map of Australia showing the redefined anthrax belt, areas predicted to support the survival of *B*. *anthracis* and anthrax outbreak locations from three historical periods. (TIF)

S3 Fig Maps of the dominant rules from each model in the best subset of GARP experiment 5 predicting presence (red rules) across the continent. Blues rules in (E) and (I) illustrate dominant single absence rules from those two rule-sets. Insets of each map indicate the presence/absence prediction from the GARP experiment.
(TIF)

S1 Table Summary accuracy statistics for 10 GARP modeling experiments. Experiments are ranked by AUC score and total omission values. (PDF)

S2 Table Number and percentages (%) of rule types from the 10 best subset models of GARP experiment 5. (PDF)
