# Supplementary figures and images for: Redefining the Australian Anthrax Belt: Modeling the Ecological Niche and Predicting the Geographic Distribution of Bacillus anthracis
Source: PLoS Negl Trop Dis. 2016 Jun 9;10(6):e0004689. doi: 10.1371/journal.pntd.0004689 (PMC4900651; doi:10.1371/journal.pntd.0004689)

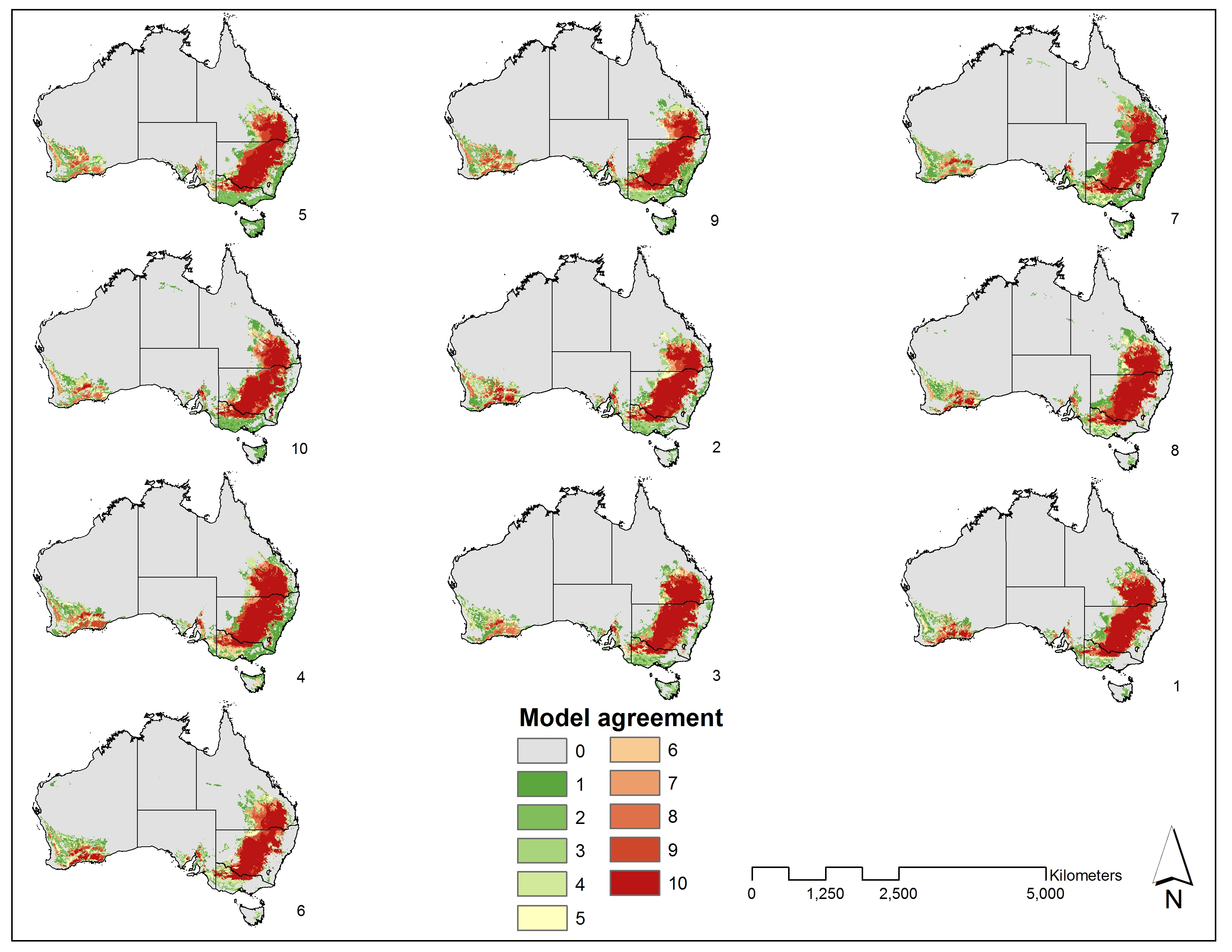

Supplement: S1 Fig — Geographic distributions are ranked from top left corner to right based on the highest AUC and lowest total omission rates listed in S1 Table. Model agreements represent the number of model(s) predicting the area to be conducive to Bacillus anthracis persistence. (TIF) [file pntd.0004689.s001.tif]

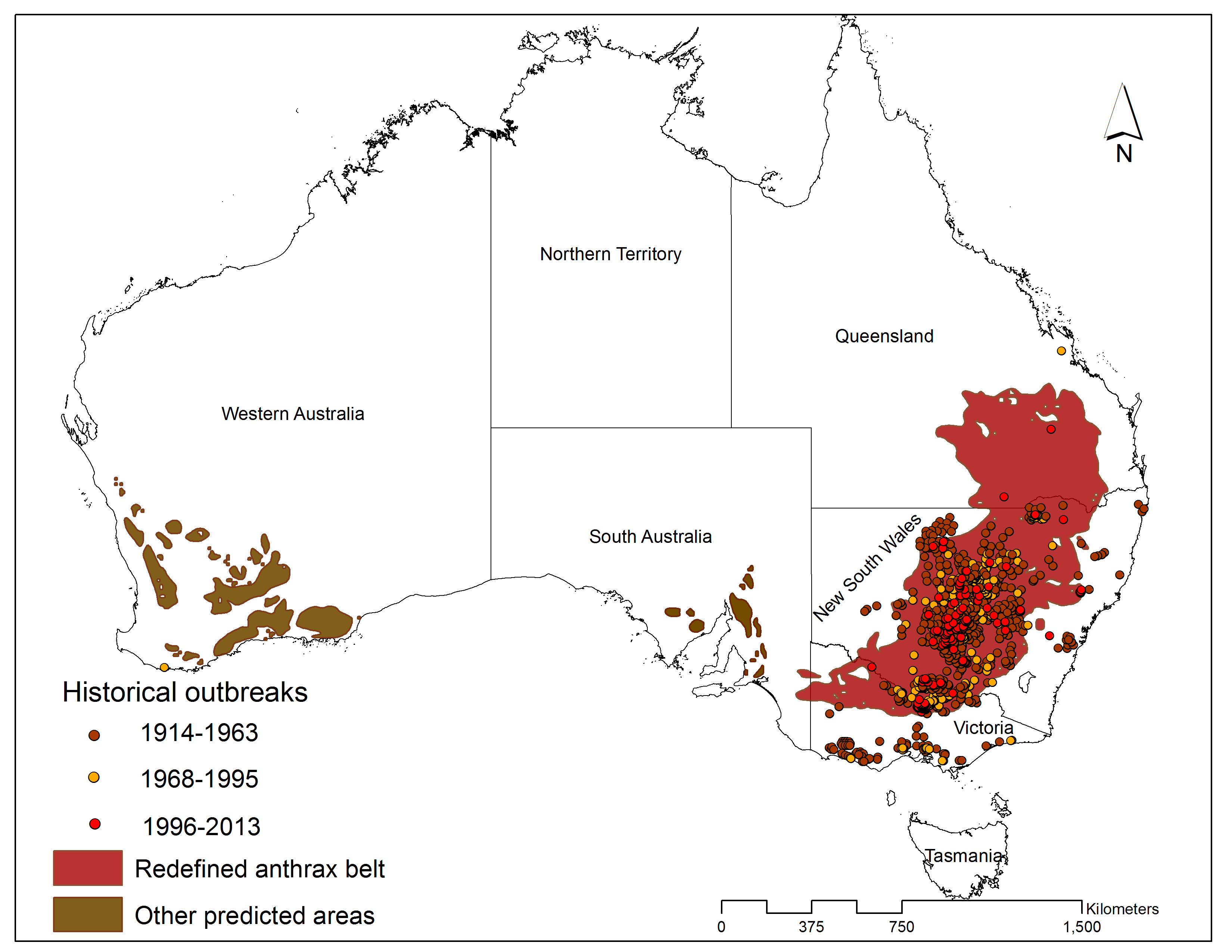

Supplement: S2 Fig — (TIF) [file pntd.0004689.s002.tif]

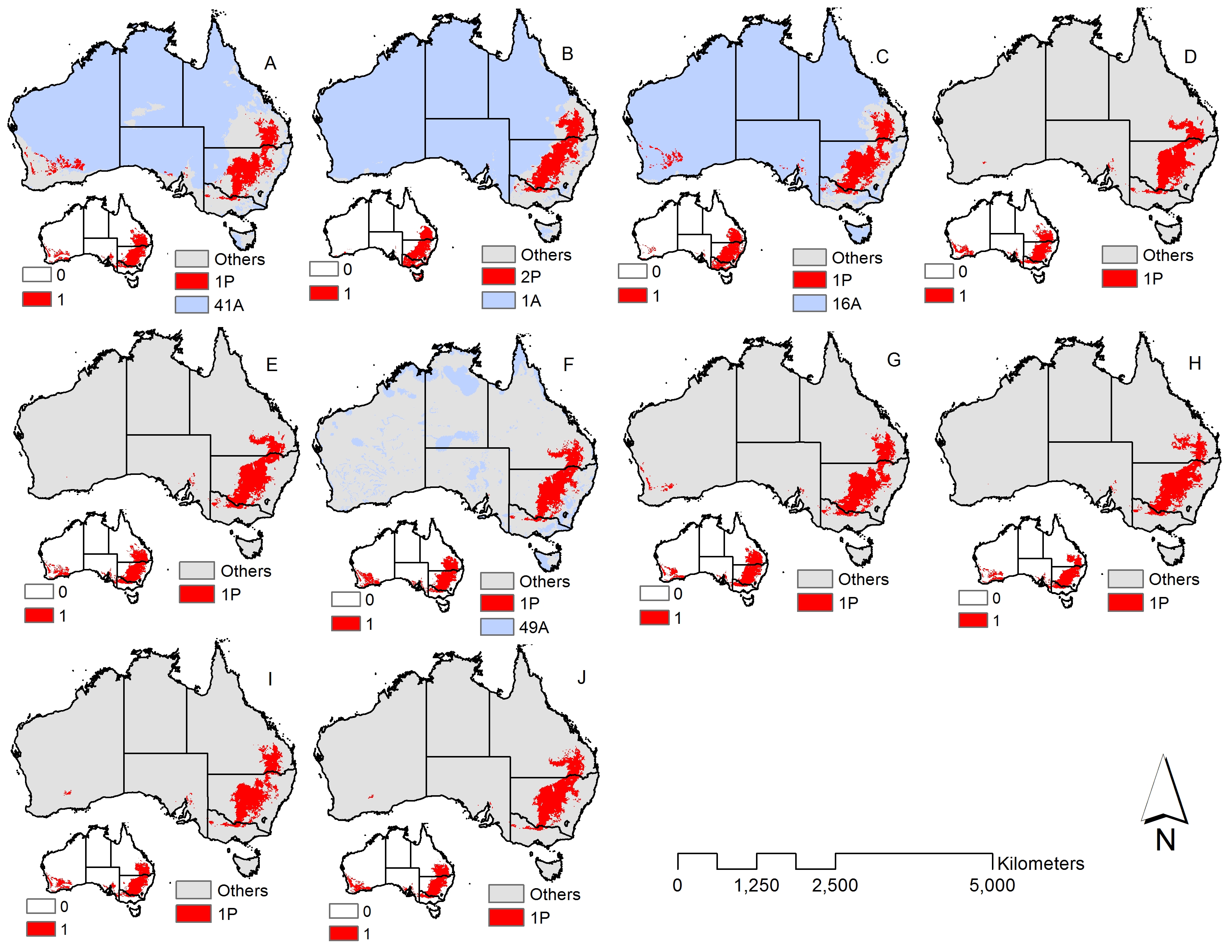

Supplement: S3 Fig — Blues rules in (E) and (I) illustrate dominant single absence rules from those two rule-sets. Insets of each map indicate the presence/absence prediction from the GARP experiment. (TIF) [file pntd.0004689.s003.tif]
