# Supplementary material for: Redefining the Australian Anthrax Belt: Modeling the Ecological Niche and Predicting the Geographic Distribution of Bacillus anthracis
Source: PLoS Negl Trop Dis. 2016 Jun 9;10(6):e0004689. doi: 10.1371/journal.pntd.0004689 (PMC4900651; doi:10.1371/journal.pntd.0004689)
Supplement: S1 Table — (PDF) [file pntd.0004689.s004.pdf]

Table S1. Summary accuracy statistics for 10 GARP modeling experiments. Experiments are ranked by AUC score and total omission values.

GARP experiments	AUC	SE	Z	Total Omission	Average Omission	Total Commission	Average Commission
Random 5	0.966	0.026	11.374	0.000	0.833	6.241	12.150
Random 9	0.962	0.027	11.227	0.000	3.333	5.966	12.503
Random 7	0.962	0.027	11.008	0.000	3.333	6.687	12.433
Random 10	0.961	0.028	11.667	0.000	1.667	6.883	11.965
Random 2	0.960	0.028	11.592	0.000	0.417	7.614	12.846
Random 8	0.960	0.028	11.794	0.000	0.417	7.598	12.690
Random 4	0.956	0.029	11.126	0.000	0.833	8.047	13.808
Random 3	0.956	0.029	11.514	0.000	2.500	7.811	12.836
Random 1	0.955	0.030	12.027	0.000	5.000	7.629	11.936
Random 6	0.918	0.039	11.848	8.333	5.278	6.770	11.784
