# Supplementary material for: Redefining the Australian Anthrax Belt: Modeling the Ecological Niche and Predicting the Geographic Distribution of Bacillus anthracis
Source: PLoS Negl Trop Dis. 2016 Jun 9;10(6):e0004689. doi: 10.1371/journal.pntd.0004689 (PMC4900651; doi:10.1371/journal.pntd.0004689)
Supplement: S2 Table — (PDF) [file pntd.0004689.s005.pdf]

Table S2. Number and percentages (%) of rule types from the 10 best subset models of GARP experiment 5.

	**Rule Set**									
**Rule Type**	**103**	**106**	**109**	**110**	**114**	**115**	**117**	**120**	**122**	**125**
Range	46(92)	48(96)	46(92)	50(100)	50(100)	49(98)	50(100)	50(100)	50(100)	50(100)
Negated Range	4(8)	1(2)	3(6)	0(0)	0(0)	1(2)	0(0)	0(0)	0(0)	0(0)
Logit	0(0)	1(2)	1(2)	0(0)	0(0)	0(0)	0(0)	0(0)	0(0)	0(0)
Atomic	0(0)	0(0)	0(0)	0(0)	0(0)	0(0)	0(0)	0(0)	0(0)	0(0)
